# Ocular Biometry OCR: a machine learning algorithm leveraging optical character recognition to extract intra ocular lens biometry measurements

**DOI:** 10.3389/frai.2024.1428716

**Published:** 2025-01-06

**Authors:** Anish Salvi, Leo Arnal, Kevin Ly, Gabriel Ferreira, Sophia Y. Wang, Curtis Langlotz, Vinit Mahajan, Chase A. Ludwig

**Affiliations:** ^1^School of Medicine, Stanford University, Palo Alto, CA, United States; ^2^Department of Medicine, Chicago Medical School at Rosalind Franklin University, North Chicago, IL, United States; ^3^Department of Medicine, Varzea Grande University Center, Várzea Grande, Brazil; ^4^Department of Ophthalmology, Byers Eye Institute, Stanford University, Palo Alto, CA, United States; ^5^Department of Radiology, Stanford University, Palo Alto, CA, United States

**Keywords:** optical character recognition, PaddleOCR, Gemini, text extraction, Lenstar, IOL master 500, IOL master 700

## Abstract

Given close relationships between ocular structure and ophthalmic disease, ocular biometry measurements (including axial length, lens thickness, anterior chamber depth, and keratometry values) may be leveraged as features in the prediction of eye diseases. However, ocular biometry measurements are often stored as PDFs rather than as structured data in electronic health records. Thus, time-consuming and laborious manual data entry is required for using biometry data as a disease predictor. Herein, we used two separate models, PaddleOCR and Gemini, to extract eye specific biometric measurements from 2,965 Lenstar, 104 IOL Master 500, and 3,616 IOL Master 700 optical biometry reports. For each patient eye, our text extraction pipeline, referred to as Ocular Biometry OCR, involves 1) cropping the report to the biometric data, 2) extracting the text via the optical character recognition model, 3) post-processing the metrics and values into key value pairs, 4) correcting erroneous angles within the pairs, 5) computing the number of errors or missing values, and 6) selecting the window specific results with fewest errors or missing values. To ensure the models’ predictions could be put into a machine learning-ready format, artifacts were removed from categorical text data through manual modification where necessary. Performance was evaluated by scoring PaddleOCR and Gemini results. In the absence of ground truth, higher scoring indicated greater inter-model reliability, assuming an equal value between models indicated an accurate result. The detection scores, measuring the number of valid values (i.e., not missing or erroneous), were Lenstar: 0.990, IOLM 500: 1.000, and IOLM 700: 0.998. The similarity scores, measuring the number of equal values, were Lenstar: 0.995, IOLM 500: 0.999, and IOLM 700: 0.999. The agreement scores, combining detection and similarity scores, were Lenstar: 0.985, IOLM 500: 0.999, and IOLM 700: 0.998. IOLM 500 was annotated for ground truths; in this case, higher scoring indicated greater model-to-annotator accuracy. PaddleOCR-to-Annotator achieved scores of detection: 1.000, similarity: 0.999, and agreement: 0.999. Gemini-to-Annotator achieved scores of detection: 1.000, similarity: 1.000, and agreement: 1.000. Scores range from 0 to 1. While PaddleOCR and Gemini demonstrated high agreement, PaddleOCR offered slightly better performance upon reviewing quantitative and qualitative results.

## Introduction

Eye structure is known to be associated with various eye diseases; for example, myopia increases the risk of cataracts, glaucoma, strabismus, myopic macular degeneration, myopic foveoschisis, and retinal detachments, while hyperopia raises the risk for uveal effusion syndrome and angle closure glaucoma. Given these connections, ocular biometry measurements—such as axial length, lens thickness, anterior chamber depth, and keratometry values—can be valuable predictors for eye conditions, alongside data from electronic health records (EHRs) and ophthalmic imaging. However, these measurements are often stored in portable document format (PDF) format rather than as structured data in EHRs, requiring manual, labor-intensive data entry for use in disease prediction.

With the global rise in myopia there has been a subsequent rise in retinal tears (RTs) retinal detachments (RDs). The severity of axial elongation impacts outcome; for example, RD is five or six times more likely in highly myopic (odds ratio > 20) as compared to low myopic (odds ratio < 4) patients (Flitcroft, 2012; Williams and Hammond, 2019). Ocular biometric measurements may serve as key features in the prediction of RDs. Optical biometers, including (Lenstar LS 900; IOLMaster 500; IOLMaster 700) provide measurements including axial length (AL), anterior chamber depth (ACD), anterior scleral thickness (AST), central corneal thickness (CCT), keratometry (K) including the flat meridian of the cornea (R1) and steep meridian of the cornea (R2), lens thickness (LT), true keratometry (TK), and white-to-white (WTW) values, that may provide insight into the development of RDs. Learning from biometry and EHR-related features, conventional machine learning (ML) algorithms may aid in predicting RT/RD progression. However, acquiring the biometry data confined within the PDF reports is a time-consuming and laborious task.

We expand the task of optical character recognition (OCR) into the biomedical domain by applying text extraction techniques to standard-of-care clinical documents. The aims of the study were 1) extracting the text of biometer data via two separate OCR models and 2) measuring the inter-model agreement. After selecting biometry measurement related PDFs from the original dataset, the text extraction algorithm was applied independently across 3 different optical biometer data types and 2 different OCR models. Performance was assessed by quantifying inter-model reliability scores between the two models’ outputs. Our text extraction algorithm, Ocular Biometry OCR, will facilitate downstream analytical tasks, including the classification of future RT/RD development via conventional ML methods.

## Materials and equipment

### Optical character recognition

OCR refers to a bevy of computational methods capable of converting textual information confined within images of typed, printed, or handwritten documents into machine encoded text. OCR models perform the tasks of text detection, localization of words within an image, and text recognition, the interpretation of the aforementioned words into machine encoded text.

Text detectors include convolutional neural networks such as DBNet (Liao et al., 2020) and DMPNet (Liu and Jin, 2017). DBNet segments out the region of text from a larger image by adaptively setting thresholds on probability maps. As a regression task, DMPNet computes quadrangles by applying sliding quadrilateral windows upon convolutional layers and Monte Carlo methods. Text recognition involves convolutional recurrent neural networks (CRNNs) in which convolutional layers generate a feature map from the input image; After, the feature maps are converted into feature sequences which pass through the recurrent layers to predict each text character in sequence (Shi et al., 2016). More novel methods have been explored like SVTR which decomposes an image into small patches referred to as character components, these components pass through hierarchical stages of mixing, merging, and combining to identify inter and intra character patterns, culminating in a linear predictor (Du et al., 2022).

Recent models have employed visual encoding strategies to interleave text and image data for performing tasks such as visual question answering, image captioning, etc. For example, PaLI generates text responses based on image and text inputs with an architecture underpinned on large pretrained encoder-decoder language models (Vaswani et al., 2017) and vision transformers with a balanced parameter share between vision and language modules for optimal performance (Chen et al., 2022). Similarly, the Flamingo family of vision language models input visual data interleaved with text to output free form text and is capable of optimizing performance after few shot learning and task specific tuning (Alayrac et al., 2022). Due to the reliance on pre-training for boosting performance, methods such as Contrastive Captioner (CoCa) have been proposed to pretrain image-text encoder-decoder foundation models with contrast and captioning objectives (Yu et al., 2022).

### Ocular Biometry OCR

**Figure 1:**
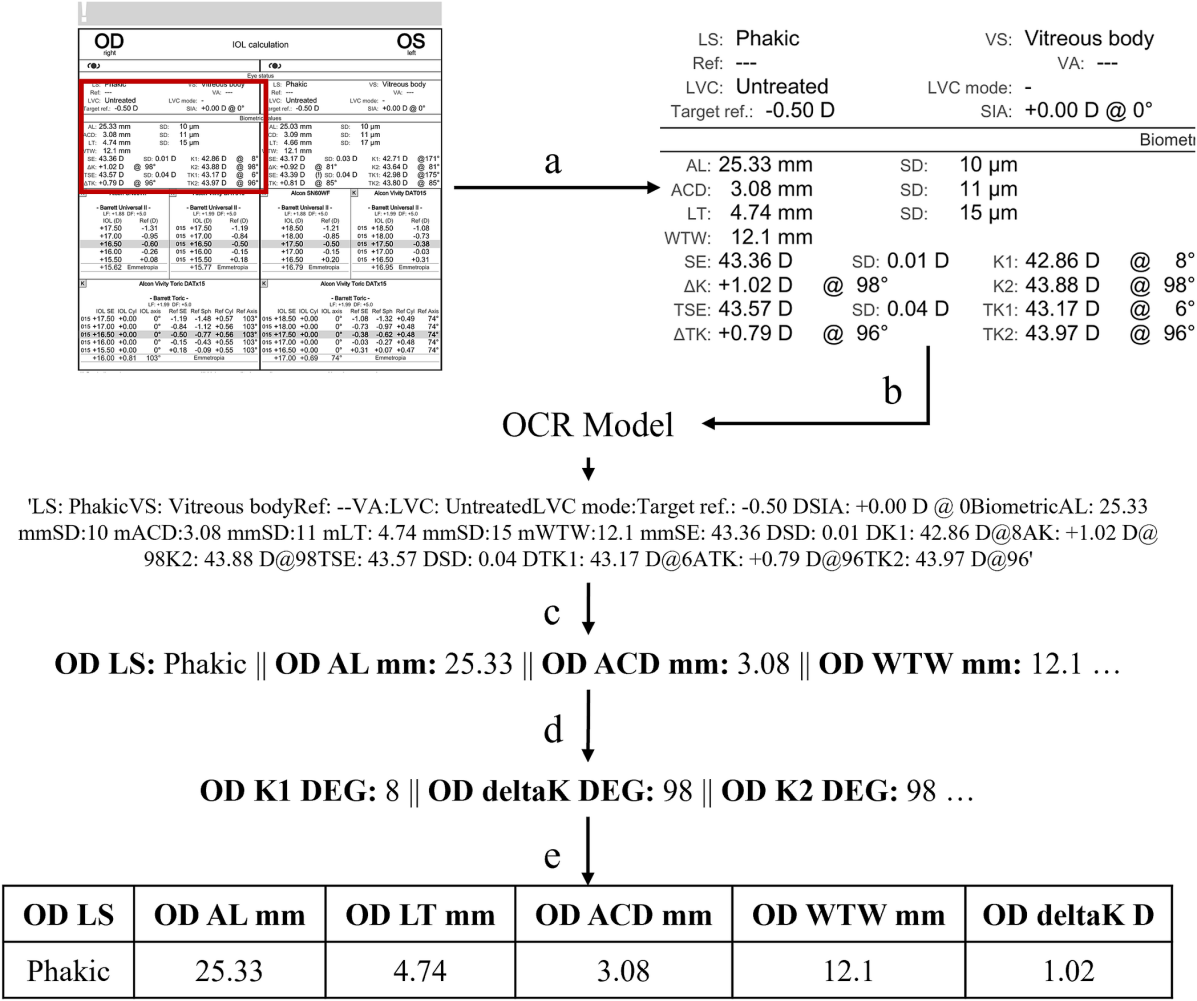
Our text extraction algorithm, Ocular Biometry OCR, where **(a)** crop biometry information; **(b)** apply PaddleOCR or Gemini; **(c)** set key-value pairs for metrics of interest; **(d)** verify and correct angles by rule checking; and **(e)** provide results in a usable, tabular, and ML ready format. This computational process can be repeated for multiple windows with the final set of key-value pairs selected based on the number of fewest missing or erroneously extracted values.

A custom text extraction algorithm, Ocular Biometry OCR, was developed to collect the clinical measurements of interest from each modality specific report (Figure 1). Prior to running the algorithm, a set of eye-specific windows is determined manually to 1) focus on the biometric measurements via cropping and 2) de-identify data and exclude protected health information (PHI). The original biometry report is cropped to the biometric data by a window. The OCR model is applied to extract all the text found within the windowed image. The clinically relevant metrics, defined beforehand, are retrieved from the model’s output and processed into a set of key-value pairs where the key is the metric and the value is the associated text or numerical quantity. If the associated value cannot be determined for a metric it is marked as an error. It was noted that identifying angles associated with K1, K2, and deltaK were particularly difficult (i.e., confusing the degree symbol for 0). To correct erroneous angles, a rules-based algorithm was applied to verify the relationship between angles; for example, angle K1 should equal angle K2 +/− 90 degrees and angle deltaK is equal to angle K2. The number of erroneous metrics was computed by summing the number of missing or undetermined metrics and the number of incorrect relationships between angles. The number of errors is collected per eye-specific window. The window with the lowest number of errors is considered the final result. The task was conducted separately for both right eye (OD) and left eye (OS). Text post processing performs optimally when the model’s prediction of metrics and values appear in the same order as in the original biometric document (i.e., right-to-left, top-to-bottom, normal English formatting). While steps are taken to address situations in which the sequence differs from expected, the ideal model would preserve the formatting. Two separate OCR models were employed.

### PaddleOCR

PaddleOCR refers to a collection of open-source models within PaddlePaddle designed for OCR tasks (Du et al., 2020; Du et al., 2021). For the study, the architecture adopted for PaddleOCR consists of two separate components: 1) text detection taken from PP-OCRv3 and 2) text recognizer taken from PP-OCRv4 (Li et al., 2022; PaddleOCR). PaddleOCR was initialized to the English language and without the angle classifier as none of the biometric data was rotated by 180 degrees. Parallel processing was implemented for faster text extraction times. Note, PaddleOCR infers upon an image.

### Gemini

Gemini refers to a suite of models developed by Google optimized for multimodal inference tasks, including video, image, text, and audio (Team et al., 2023). The Gemini architecture is based upon transformer decoders (Vaswani et al., 2017) and utilizes multi-query attention (Shazeer, 2019). As part of the study, Gemini Pro Vision was implemented with a temperature of 0.1 and maximum output tokens of 2048 (default). Given the task of text extraction, a lower temperature value was selected for a more deterministic and less creative response as compared to the default value of 0.4. Parallel processing was not implemented for Gemini text extraction because the number of Gemini inference requests per second was rate limited. Note, Gemini infers upon an image and prompt.

## Methods

**Figure 2:**
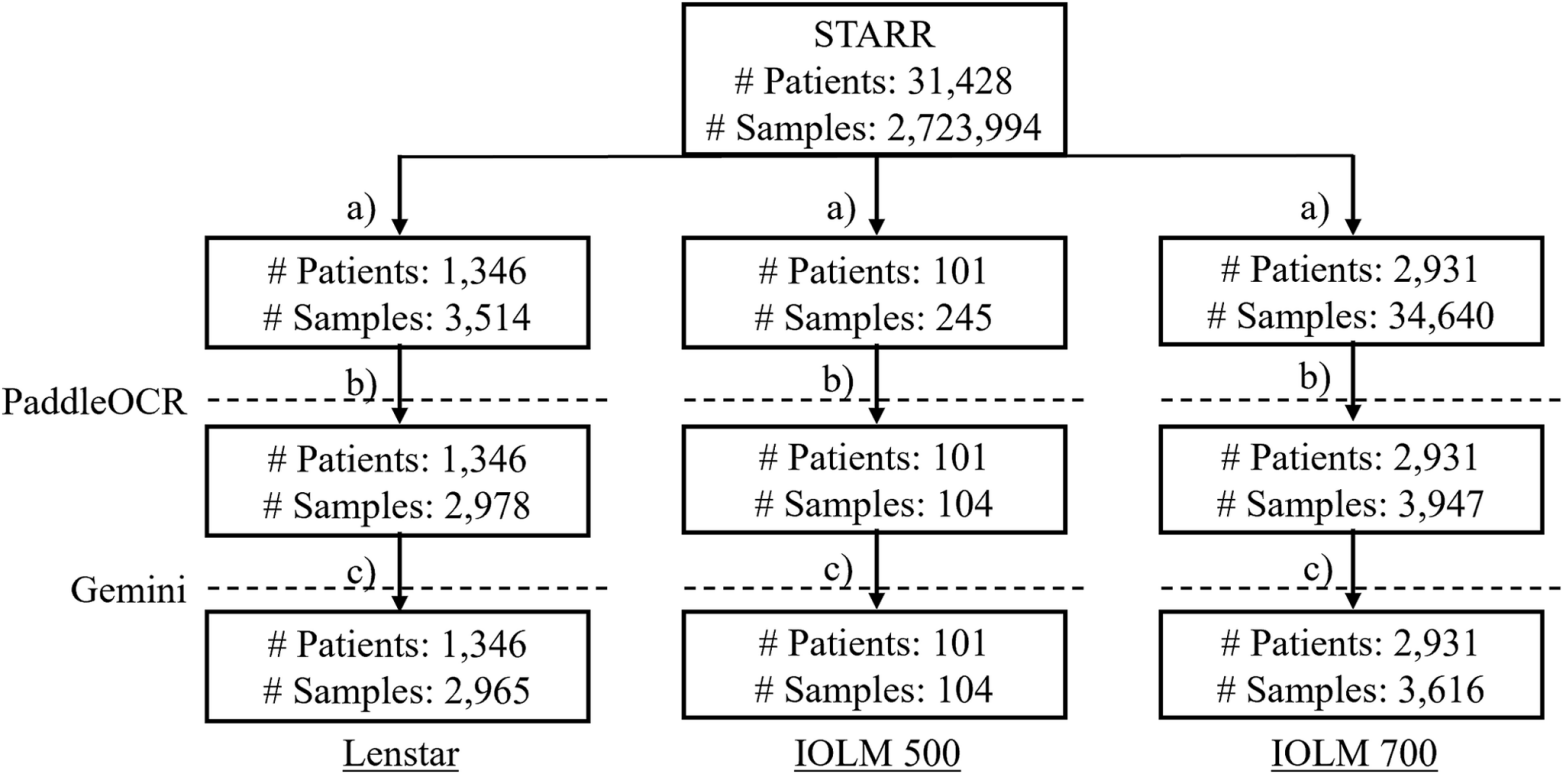
The data inclusion workflow where **(a)** subset the biometer modality; **(b)** drop duplicate data entries, keep files with the relevant PDF (only IOLM 700), drop duplicate samples, drop samples without reports, drop duplicate samples by file-to-file comparison, drop duplicate samples with identical cropped biometry information; **(c)** drop duplicate PaddleOCR metrics of interest and output, and interpret final dataset by Gemini.

PaddleOCR was selected as representative of the status quo of publicly available OCR algorithms whereas Gemini was chosen to represent more novel and commercially available technology. Biometric reports were retrieved from the Stanford Research Repository (STARR), Stanford’s Clinical Data Warehouse, and securely stored in a dedicated storage bucket within a protected computing environment. The original cohort contained 31,428 patients and 2,723,994 samples. Optical biometer specific subgroups were identified after addressing common clinical practices which may affect results (duplicated data, missing entries, etc.). The data inclusion workflow ensured quality documents (no missingness or repetitiveness). The rationale was that identical inputs lead to identical outputs and would either lack meaningfulness or skew performance results (Figure 2). For each subgroup, several key metrics were identified for text extraction.

### Lenstar dataset

For Lenstar text extraction, the biometric information of interest includes: LS, AL (mm), CCT (um), AD (mm), ACD (mm), LT (mm), R1 (D + angle), R2 (D + angle), R (D), AST (D + angle), n, WTW (mm), and Target Refraction.

For Lenstar, text extraction was performed by PaddleOCR for the 2,978 reports identified for 1,346 patients. If between samples, the metrics of interest and model output produced by PaddleOCR were duplicated, the samples were removed. Text extraction via Gemini was performed on the remaining 2,965 reports for 1,346 patients. In an effort to reduce unordered text and specific to the Lenstar modality, the same image was prompted twice for text extraction per sample. The first prompt, from which LS was extracted, was: “Extract all the text in the entire image and keep the formatting”. The second prompt to extract the remaining data was: “Extract metrics in the format ‘AL [mm]’ x, ‘CCT [um]’ x, ‘AD [mm]’ x, ‘ACD [mm]’ x, ‘LT [mm]’ x, and ‘Target Refraction: ‘x where x is the value. If x is missing, skip the value. Extract metrics in the format, ‘R1 [mm/D/°]’ x ‘/’ x ‘@’ x, ‘R2 [mm/D/°]’ x ‘/’ x ‘@’ x, ‘R [mm/D]’ x ‘/’ x, ‘A ST [D/°]’ x ‘@’ x, ‘n ‘x, ‘WTW [mm]’ x where x is the full line of all the text immediately to the right of the metric. If the text immediately to the right of the metric is blank, skip the metric. Ignore the values for IOL and Eye. No bullet points. No colons. No superscripts.”; The reason the ‘AST’ was written as ‘A ST’ was because within certain documents it appears as such, in an effort to increase algorithmic robustness.

### IOLM 500 dataset

For IOLM 500 text extraction, the biometric information of interest includes: LS, AL (mm), ACD (mm), WTW (mm), K1 (D + angle), K2 (D + angle), deltaK (D + angle).

For IOLM 500, text extraction was performed by PaddleOCR for the 104 reports identified for 101 patients. As no samples were found to be duplicated according to the metrics of interest and model output of PaddleOCR, no samples were removed. Text extraction via Gemini was performed on all 104 reports for 101 patients. Gemini was prompted to “Extract all the text in the image from top to bottom. If there is no text, extract nothing. No dashes or colons.” for this exercise.

### IOLM 700 dataset

For IOLM 700 text extraction, the biometric information of interest includes: LS, vitreous status (VS), laser vision correction (LVC), Target ref., surgically induced astigmatism (SIA) (D + angle), AL (mm, SD), ACD (mm, SD), LT (mm, SD), WTW (mm), spherical equivalent (SE) (D, SD), K1 (D + angle), deltaK (D + angle), K2 (D + angle), TSE (D, SD), TK1 (D + angle), deltaTK (D + angle), and TK2 (D + angle). Unlike Lenstar and IOLM 500, the biometric information for IOLM 700 was not limited to the first page of the patient report and had a wider variation in formatting.

For IOLM 700, text extraction was performed by PaddleOCR for the 3,947 reports identified for 2,931 patients. If between samples, the PaddleOCR metrics of interest and output were duplicated, the samples were removed. Text extraction via Gemini was performed on the remaining 3,616 reports for 2,931 patients. Gemini was prompted to “Extract all the text in the entire image and keep the original formatting. Include the standard deviations (SD) and angles (@). Denote standard deviation as SD not +/−.” for this exercise.

### Performance evaluation

For text data (LS, VS, LVC), the output quantities were manually processed for artifacts, modifying results to within the known categorical values present for each optical biometer subgroup. In other words, the unique responses per text metric were viewed and through human intervention, a rules-based algorithm was developed to automatically match certain text outputs with the expected result. For example, if the text extraction algorithm predicted “Phakica” for LS with the letter “a” clearly being an artifact, the final result would be considered “Phakic”.

In the absence of ground truth data, four separate metrics were identified to assess detection, similarity, and agreement and served as an indicator of inter-model reliability, also assuming that equal values indicated a more accurate result. This included detection, similarity, and agreement scores which range from 0 to 1 with a higher score indicating fewer differences and greater inter-model reliability between the values extracted via PaddleOCR to those extracted by Gemini. In addition, the Cohen-Kappa statistic, which ranges from −1 (i.e., inverse prediction) to 1 (i.e., perfect prediction), was computed to measure inter-model reliability between PaddleOCR and Gemini for the task of detection (Cohen, 1960). The four performance measures were applied across all optical biometers.

Given that inter-model reliability quantified performance, as opposed to ground truth data, several limitations include: 1) the same, yet incorrect predictions for both models and 2) a specific formatting style of a model’s prediction was not accounted for as part of the post-processing strategy causing the relevant text to be extracted incorrectly. To gauge these mistakes, an annotator manually collected IOLM 500 biometric information for reference as ground truth.

## Results

(1) $$\mathrm{Detectio}n_{\mathrm{Score}}=\frac{\mathrm{Detectio}n_{-/-}+\mathrm{Detectio}n_{+/+}}{\mathrm{Detectio}n_{\mathrm{All}}}$$

Detection score between PaddleOCR & Gemini.

Detection indicated that the text algorithm retrieved a valid value for a specific metric. The positive and negative detections were counted for each model. A positive detection (+) indicated that a valid value was predicted for the metric whereas a negative detection (−) indicated that an erroneous or missing value was associated with the metric. The detection score was then computed as the ratio of all positive and negative detections (i.e., between PaddleOCR and Gemini) summed over the total number of detections (Equation 1).


(2) $$\begin{array}{l} D_{\mathrm{Cohen}-\mathrm{Kappa}}\\ =\frac{2*\left[\left(D_{+/+}*D_{-/-}\right)+\left(D_{+/-}*D_{-/+}\right)\right]}{\left(D_{+/+}+D_{-/+}\right)*\left(D_{-/+}+D_{-/-}\right)+\left(D_{+/+}+D_{+/-}\right)*\left(D_{+/-}+D_{-/-}\right)} \end{array}$$

Cohen-Kappa statistic where *D* indicates the detection quantity for PaddleOCR & Gemini.

The Cohen-Kappa statistic, leveraging the appropriate detection quantities for PaddleOCR and Gemini, was also calculated (Equation 2).


(3) $$\mathrm{Similarity}=\{\begin{array}{c} 1\;\mathrm{if}\;{\hat{y}}_{\mathrm{PaddleOCR}}={\hat{y}}_{\mathrm{Gemini}}\;\\ 0\;\mathrm{if}\;{\hat{y}}_{\mathrm{PaddleOCR}}\neq {\hat{y}}_{\mathrm{Gemini}} \end{array}$$

Nominal similarity where ŷ indicates a predicted value.

The similarity indicated the number of metrics for which the values associated with all positive values (i.e., between PaddleOCR and Gemini) are equal or different. In other words, if both values were successfully retrieved for a specific sample and metric in both models, the similarity measures if the two values are equal to each other (Equation 3). The similarity score is computed as the ratio of all equal values (i.e., where the result of PaddleOCR is the same as that of Gemini) divided by the total number of all positive detections (i.e., where the results of PaddleOCR and Gemini were successfully retrieved).


(4) $$\mathrm{Agreemen}t_{\mathrm{Score}}=\frac{\mathrm{Detectio}n_{-/-}+\mathrm{Similarit}y_{+}}{\mathrm{Detectio}n_{\mathrm{all}}}$$

Observed agreement between PaddleOCR & Gemini.

The agreement score was computed as the ratio of the equivalent values and all negative detections (i.e., between PaddleOCR and Gemini) summed over the total number of detections (Equation 4). The agreement score combines the detection and similarity scores to indicate overall inter-model agreement and performance of the two models.

### Lenstar

Indicative of inter-model reliability, the average scores were 0.990 for detection, 0.995 for similarity, and 0.985 for agreement while the average Cohen-Kappa statistic was 0.629 for detection (Table 1).

**Table 1:** Inter-model reliability between PaddleOCR and Gemini per Lenstar metric across OD and OS.

Lenstar	Detection						Similarity			Agreement score
Metric	PaddleOCR (−) & Gemini (−)	PaddleOCR (−) & Gemini (+)	PaddleOCR (+) & Gemini (−)	PaddleOCR (+) & Gemini (+)	Cohen Kappa	Score	(=)	(≠)	Score	
ACD mm	245	57	1	5,627	0.889	0.990	5,536	91	0.984	0.975
AD mm	255	47	8	5,620	0.898	0.991	5,549	71	0.987	0.979
AL mm	5	74	0	5,851	0.118	0.988	5,850	1	1.000	0.987
AST D	71	2	7	5,850	0.940	0.998	5,788	62	0.989	0.988
AST DEG	118	34	2	5,776	0.865	0.994	5,757	19	0.997	0.991
CCT um	7	47	0	5,876	0.228	0.992	5,857	19	0.997	0.989
LS	0	0	173	5,757	NA	0.971	5,757	0	1.000	0.971
LT mm	299	58	2	5,571	0.903	0.990	5,570	1	1.000	0.990
R D	44	1	54	5,831	0.611	0.991	5,814	17	0.997	0.988
R1 D	48	17	19	5,846	0.724	0.994	5,824	22	0.996	0.990
R1 DEG	131	21	4	5,774	0.911	0.996	5,755	19	0.997	0.993
R2 D	51	17	16	5,846	0.753	0.994	5,819	27	0.995	0.990
R2 DEG	118	34	2	5,776	0.865	0.994	5,757	19	0.997	0.991
Target ref. D	91	51	129	5,659	0.488	0.970	5,652	7	0.999	0.968
WTW mm	187	50	2	5,691	0.873	0.991	5,680	11	0.998	0.989
n	0	0	23	5,907	NA	0.996	5,838	69	0.988	0.984
Average					0.629	0.990			0.995	0.985

Lens status (LS), axial length (AL), central corneal thickness (CCT), aqueous depth (AD), anterior chamber depth (ACD), lens thickness (LT), flat meridian of the cornea (R1), steep meridian of the cornea (R2), average of R1 and R2 (R), anterior scleral thickness (AST), white-to-white (WTW), target refraction (Target ref.), diopters (D), degrees (DEG), and millimeters (mm).

### IOLM 500

Indicative of inter-model reliability, the average scores were 1.000 for detection, 0.999 for similarity, and 0.999 for agreement while the average Cohen-Kappa statistic was 1.000 for detection (Table 2). Comparing PaddleOCR to ground truth, the average scores were 1.000 for detection, 0.999 for similarity, and 0.999 for agreement while the average Cohen-Kappa statistic was 1.000 for detection (Table 3a). Comparing Gemini to ground truth, the average scores were 1.000 for detection, 1.000 for similarity, and 1.000 for agreement while the average Cohen-Kappa statistic was 1.000 for detection (Table 3b).

**Table 2:** Inter-model reliability between PaddleOCR and Gemini per IOLM 500 metric across OD and OS.

IOLM 500	Detection						Similarity			Agreement score
Metric	PaddleOCR (−) & Gemini (−)	PaddleOCR (−) & Gemini (+)	PaddleOCR (+) & Gemini (−)	PaddleOCR (+) & Gemini (+)	Cohen Kappa	Score	(=)	(≠)	Score	
ACD mm	56	0	0	152	1.000	1.000	152	0	1.000	1.000
AL mm	22	0	0	186	1.000	1.000	186	0	1.000	1.000
K1 D	19	0	0	605	1.000	1.000	605	0	1.000	1.000
K1 DEG	19	0	0	605	1.000	1.000	603	2	0.997	0.997
K2 D	19	0	0	605	1.000	1.000	605	0	1.000	1.000
K2 DEG	19	0	0	605	1.000	1.000	603	2	0.997	0.997
LS	2	0	0	206	1.000	1.000	206	0	1.000	1.000
WTW mm	40	0	0	376	1.000	1.000	376	0	1.000	1.000
deltaK D	19	0	0	605	1.000	1.000	605	0	1.000	1.000
deltaK DEG	19	0	0	605	1.000	1.000	603	2	0.997	0.997
Average					1.000	1.000			0.999	0.999

Lens status (LS), axial length (AL), flat meridian of the cornea (K1), steep meridian of the cornea (K2), subtraction of K1 from K2 (delta K), anterior chamber depth (ACD), white-to-white (WTW), diopters (D), degrees (DEG), and millimeters (mm).

**Table 3:** Performance computed between **a)** Annotator and PaddleOCR and **b)** Annotator and Gemini per IOLM 500 metric across OD and OS.

IOLM 500										
a)	Detection						Similarity			Agreement score
Metric	Annotator (−) & PaddleOCR (−)	Annotator (−) & PaddleOCR (+)	Annotator (+) & PaddleOCR (−)	Annotator (+) & PaddleOCR (+)	Cohen Kappa	Score	(=)	(≠)	Score	
ACD mm	56	0	0	152	1.000	1.000	152	0	1.000	1.000
AL mm	22	0	0	186	1.000	1.000	186	0	1.000	1.000
K1 D	19	0	0	605	1.000	1.000	605	0	1.000	1.000
K1 DEG	19	0	0	605	1.000	1.000	603	2	0.997	0.997
K2 D	19	0	0	605	1.000	1.000	605	0	1.000	1.000
K2 DEG	19	0	0	605	1.000	1.000	603	2	0.997	0.997
LS	2	0	0	206	1.000	1.000	206	0	1.000	1.000
WTW mm	40	0	0	376	1.000	1.000	376	0	1.000	1.000
deltaK D	19	0	0	605	1.000	1.000	605	0	1.000	1.000
deltaK DEG	19	0	0	605	1.000	1.000	603	2	0.997	0.997
Average					1.000	1.000			0.999	0.999

b)	Detection						Similarity			Agreement Score
Metric	Annotator (−) & Gemini (−)	Annotator (−) & Gemini (+)	Annotator (+) & Gemini (−)	Annotator (+) & Gemini (+)	Cohen Kappa	Score	(=)	(≠)	Score	
ACD mm	56	0	0	152	1.000	1.000	152	0	1.000	1.000
AL mm	22	0	0	186	1.000	1.000	186	0	1.000	1.000
K1 D	19	0	0	605	1.000	1.000	605	0	1.000	1.000
K1 DEG	19	0	0	605	1.000	1.000	605	0	1.000	1.000
K2 D	19	0	0	605	1.000	1.000	605	0	1.000	1.000
K2 DEG	19	0	0	605	1.000	1.000	605	0	1.000	1.000
LS	2	0	0	206	1.000	1.000	206	0	1.000	1.000
WTW mm	40	0	0	376	1.000	1.000	376	0	1.000	1.000
deltaK D	19	0	0	605	1.000	1.000	605	0	1.000	1.000
deltaK DEG	19	0	0	605	1.000	1.000	605	0	1.000	1.000
Average					1.000	1.000			1.000	1.000

Lens status (LS), axial length (AL), flat meridian of the cornea (K1), steep meridian of the cornea (K2), subtraction of K1 from K2 (delta K), anterior chamber depth (ACD), white-to-white (WTW), diopters (D), degrees (DEG), and millimeters (mm).

### IOLM 700

Indicative of inter-model reliability, the average scores were 0.998 for detection, 0.999 for similarity, and 0.998 for agreement while the average Cohen-Kappa statistic was 0.943 for detection (Table 4).

**Table 4:** Inter-model reliability between PaddleOCR and Gemini per IOLM 700 metric across OD and OS.

IOLM 700	Detection						Similarity			Agreement score
Metric	PaddleOCR (−) & Gemini (−)	PaddleOCR (−) & Gemini (+)	PaddleOCR (+) & Gemini (−)	PaddleOCR (+) & Gemini (+)	Cohen Kappa	Score	(=)	(≠)	Score	
ACD mm	233	0	1	6,998	0.998	1.000	6,998	0	1.000	1.000
AL mm	178	0	1	7,053	0.997	1.000	7,053	0	1.000	1.000
K1 D	150	0	9	7,073	0.970	0.999	7,072	1	1.000	0.999
K1 DEG	150	0	9	7,073	0.970	0.999	7,035	38	0.995	0.994
K2 D	151	0	13	7,068	0.958	0.998	7,067	1	1.000	0.998
K2 DEG	146	5	0	7,081	0.983	0.999	7,043	38	0.995	0.994
LS	15	0	12	7,205	0.714	0.998	7,204	1	1.000	0.998
LT mm	251	0	0	6,981	1.000	1.000	6,981	0	1.000	1.000
LVC	97	1	12	7,122	0.936	0.998	7,122	0	1.000	0.998
SD	5,075	0	14	31,071	0.998	1.000	31,052	19	0.999	0.999
SE D	150	0	6	7,076	0.980	0.999	7,074	2	1.000	0.999
SIA D	218	0	4	7,010	0.991	0.999	7,010	0	1.000	0.999
SIA DEG	218	0	4	7,010	0.991	0.999	7,000	10	0.999	0.998
TK1 D	2,185	1	4	5,042	0.998	0.999	5,040	2	1.000	0.999
TK1 DEG	2,186	1	4	5,041	0.998	0.999	5,040	1	1.000	0.999
TK2 D	2,187	0	5	5,040	0.998	0.999	5,040	0	1.000	0.999
TK2 DEG	2,177	10	2	5,043	0.996	0.998	5,042	1	1.000	0.998
TSE D	2,186	0	3	5,043	0.999	1.000	5,043	0	1.000	1.000
Target ref. D	218	0	23	6,991	0.948	0.997	6,991	0	1.000	0.997
VS	18	1	85	7,128	0.292	0.988	7,128	0	1.000	0.988
WTW mm	118	0	5	7,109	0.979	0.999	7,108	1	1.000	0.999
deltaK D	252	0	47	6,933	0.911	0.994	6,932	1	1.000	0.993
deltaK DEG	146	5	0	7,081	0.983	0.999	7,043	38	0.995	0.994
deltaTK D	2,263	10	2	4,957	0.996	0.998	4,956	1	1.000	0.998
deltaTK DEG	2,177	10	2	5,043	0.996	0.998	5,042	1	1.000	0.998
Average					0.943	0.998			0.999	0.998

Lens status (LS), vitreous status (VS), laser vision correction (LVC), surgically induced astigmatism (SIA), target refraction (Target ref.), axial length (AL), anterior chamber depth (ACD), lens thickness (LT), white-to-white (WTW), spherical equivalent of corneal power according to keratometry (SE), flat meridian of the cornea (K1), steep meridian of the cornea (K2), subtraction of K1 from K2 (delta K), true flat meridian of the cornea (TK1), true steep meridian of the cornea (TK2), subtraction of TK1 from TK2 (delta TK), and spherical equivalent of corneal power according to TK (TSE), standard deviation associated with individual metrics (SD), diopters (D), degrees (DEG), and millimeters (mm).

## Discussion

### Lenstar

Assessing the detection scores of PaddleOCR and Gemini, the models heavily disagreed for LS and Target Refraction. There were various circumstances which resulted in the disputed samples for LS. These challenges include the: 1) Gemini request for the first prompt used to extract LS appears to have failed to run, 2) useful biometric information necessary for proper text extraction was cut out at the window edges, and 3) value for LS was in the wrong location for proper text extraction not in right-to-left, top-to-bottom, normal English formatting. When checking the same for Target Refraction, Gemini did not retrieve the relevant metric and value for the second prompt.

The average Cohen-Kappa statistic indicated substantial agreement (0.61–0.80) for detection, though there was wide variation in performance by metric owing to high numbers of disputed samples (i.e., false negatives and false positives overwhelming true negatives) in certain cases. For LS and n, the Cohen Kappa statistic could not be determined because there were no all negative detections for those specific metrics.

**Figure 3:**
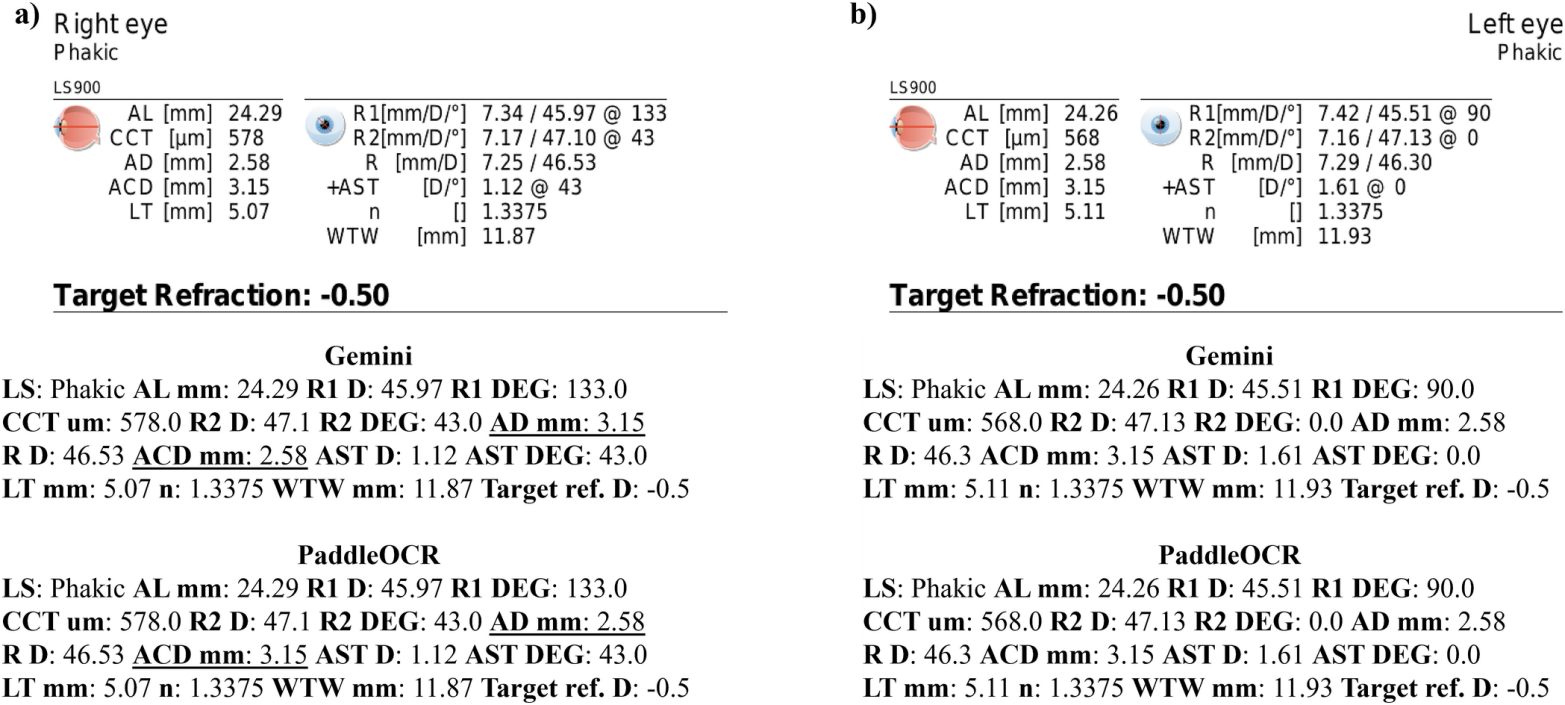
Ocular Biometry OCR for Lenstar with results for the **(a)** right eye and **(b)** left eye. For Gemini, the right eye had swapped values for AD mm and ACD mm. Lens status (LS), axial length (AL), central corneal thickness (CCT), aqueous depth (AD), anterior chamber depth (ACD), lens thickness (LT), flat meridian of the cornea (R1), steep meridian of the cornea (R2), average of R1 and R2 (R), anterior scleral thickness (AST), and white-to-white (WTW). Target refraction is listed at the bottom.

**Figure 4:**
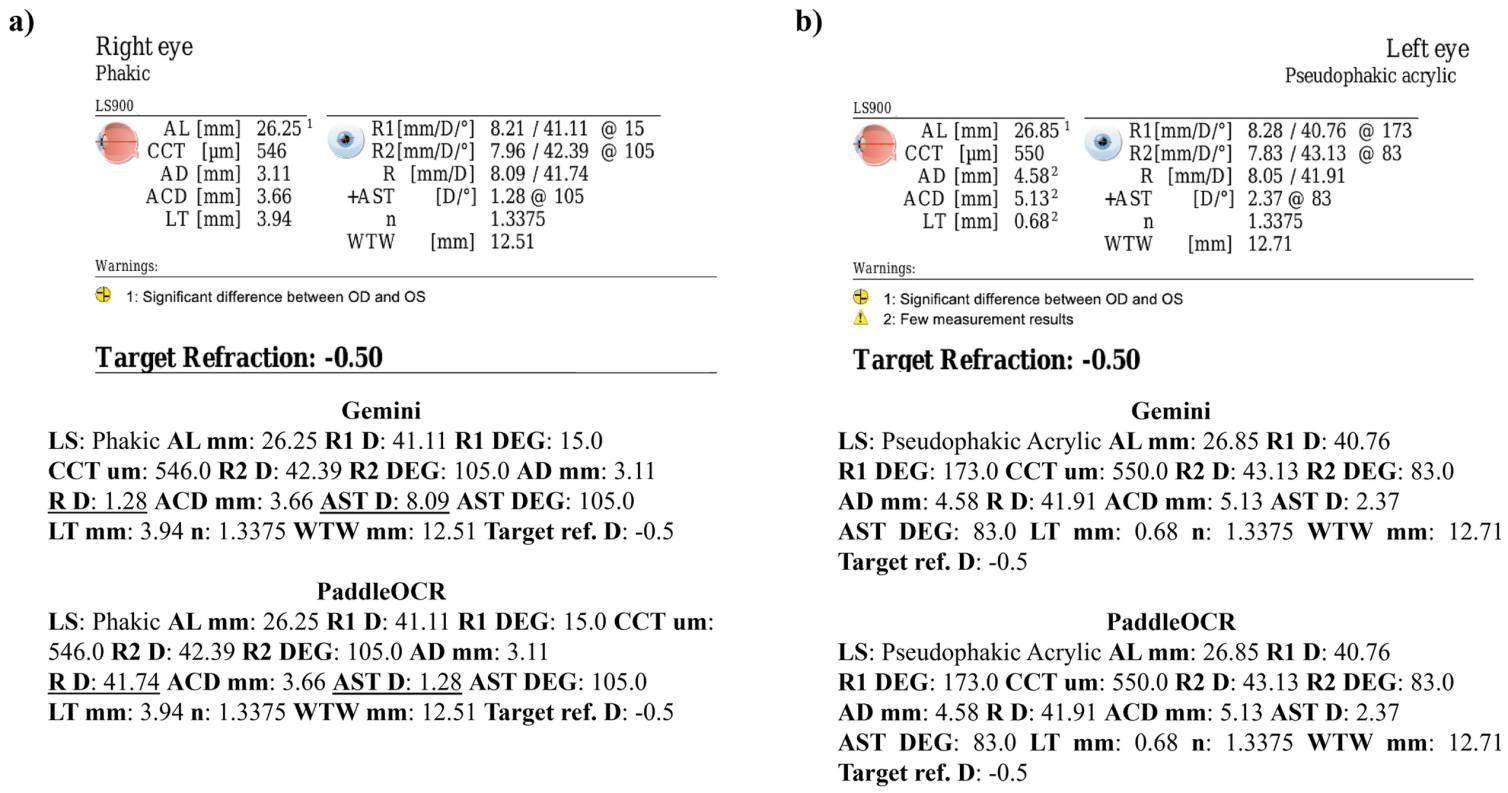
Ocular Biometry OCR for Lenstar with results for the **(a)** right eye and **(b)** left eye. For Gemini, the right eye had swapped values for R D and AST D. Lens status (LS), axial length (AL), central corneal thickness (CCT), aqueous depth (AD), anterior chamber depth (ACD), lens thickness (LT), flat meridian of the cornea (R1), steep meridian of the cornea (R2), average of R1 and R2 (R), anterior scleral thickness (AST), and white-to-white (WTW). Target refraction is listed at the bottom.

**Figure 5:**
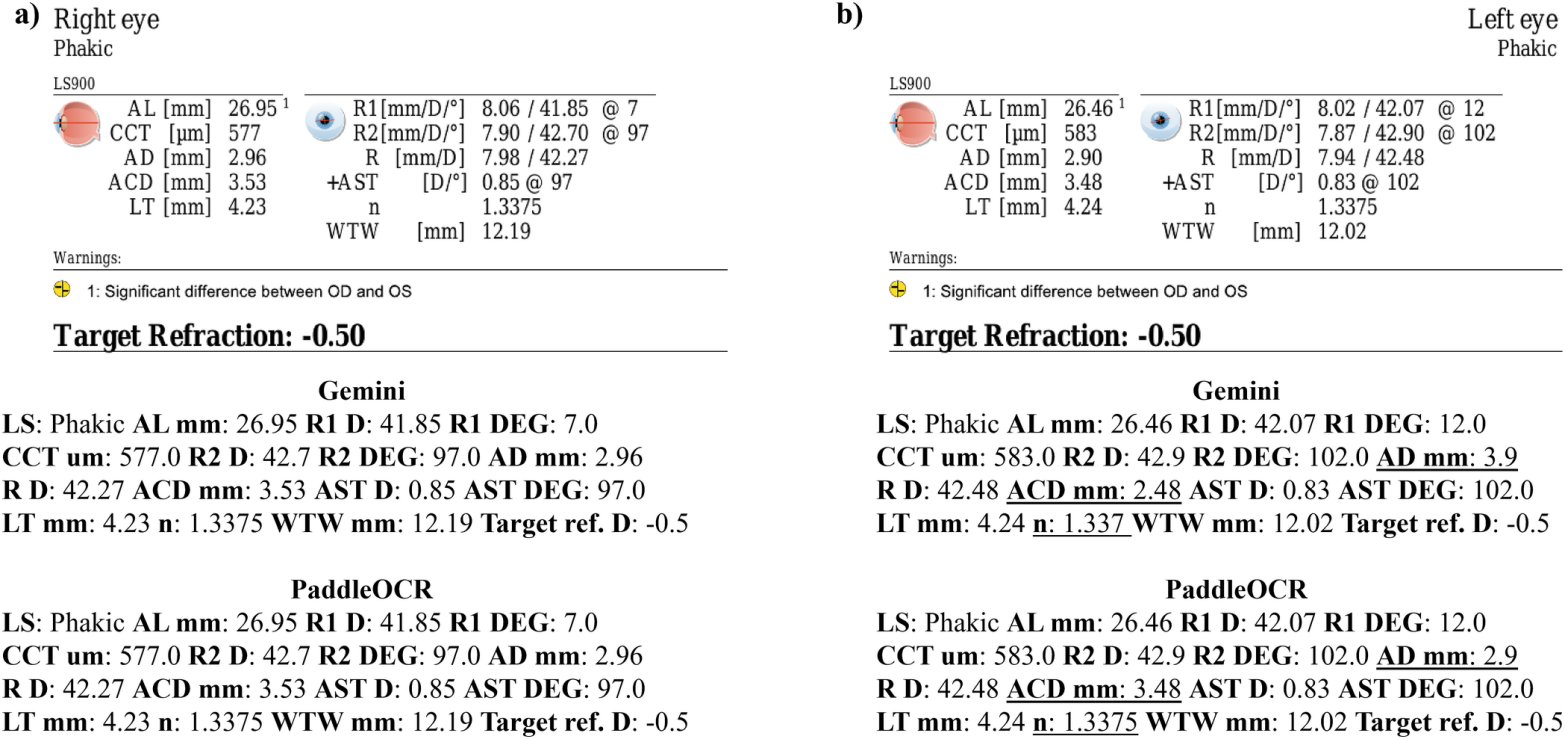
Ocular Biometry OCR for Lenstar with results for the **(a)** right eye and **(b)** left eye. For Gemini, the left eye had swapped digits for AD mm and ACD mm as well as the last digit of *n* being missing. Lens status (LS), axial length (AL), central corneal thickness (CCT), aqueous depth (AD), anterior chamber depth (ACD), lens thickness (LT), flat meridian of the cornea (R1), steep meridian of the cornea (R2), average of R1 and R2 (R), anterior scleral thickness (AST), and white-to-white (WTW). Target refraction is listed at the bottom.

Reviewing the similarity scores of PaddleOCR and Gemini, it was unveiled that ACD mm, AD mm, AST D, and *n* had some of the lowest scores. There were several Gemini related errors in this regard, issues included: 1) swapping the value of AD mm and ACD mm, 2) mistaking the value of R D for AST D, 3) providing AST degrees (DEG) twice, 4) missing a digit for n, and 5) mistaking AST D for *n* (Figures 3–5). Across all three optical biometers, PaddleOCR and Gemini had the lowest agreement score for Lenstar, likely owing to the relative complexity of the biometric data as compared to the other biometer documents.

### IOLM 500

**Figure 6:**
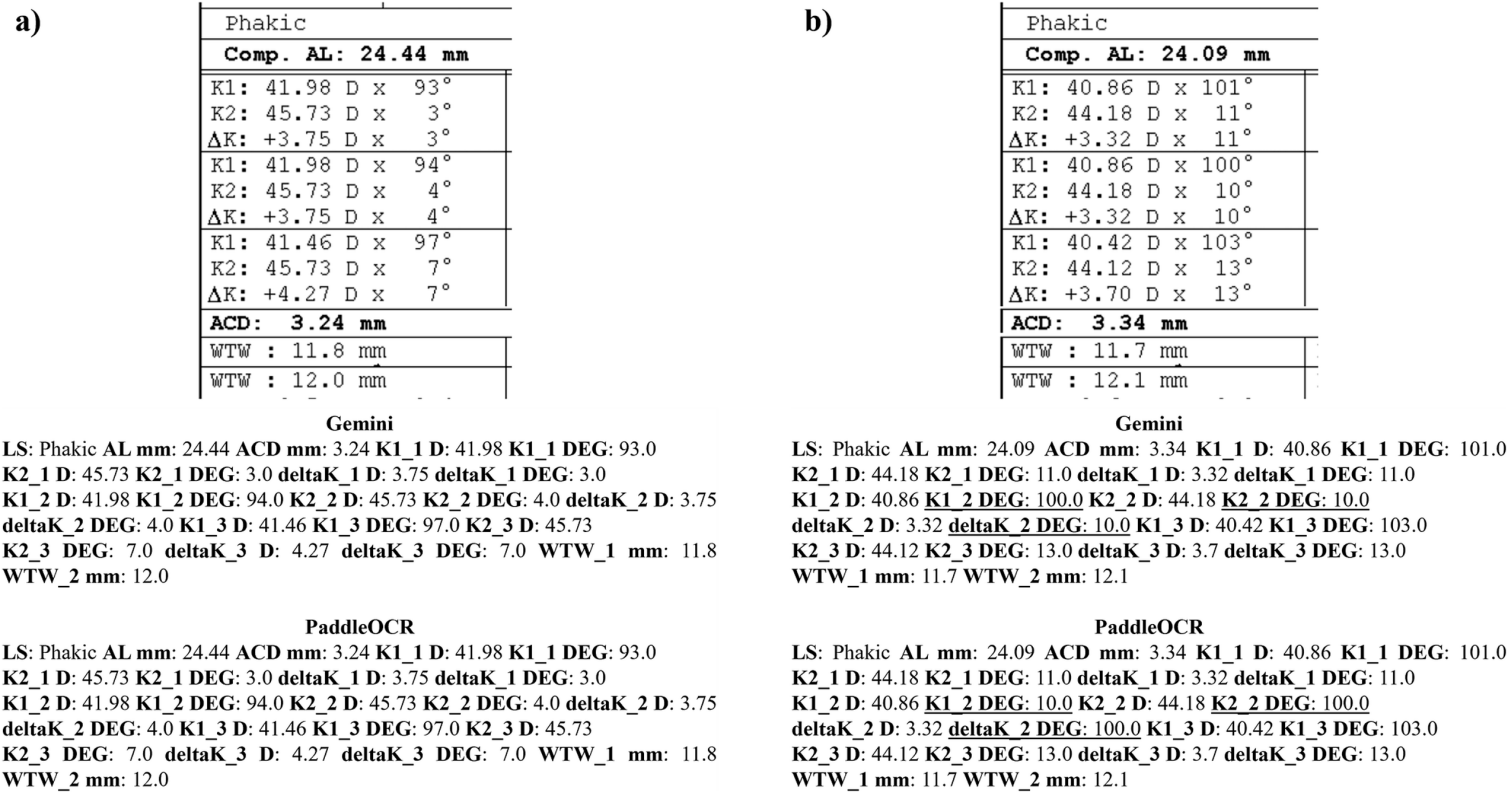
Ocular Biometry OCR for IOLM 500 with results for the **(a)** right eye and **(b)** left eye. For PaddleOCR, the left eye had swapped values for K1_2 DEG, K2_2 DEG, and deltaK_2 DEG. Axial length (AL), flat meridian of the cornea (K1), steep meridian of the cornea (K2), subtraction of K1 from K2 (delta K), anterior chamber depth (ACD), and white-to-white (WTW). Lens status (LS) is at the top.

**Figure 7:**
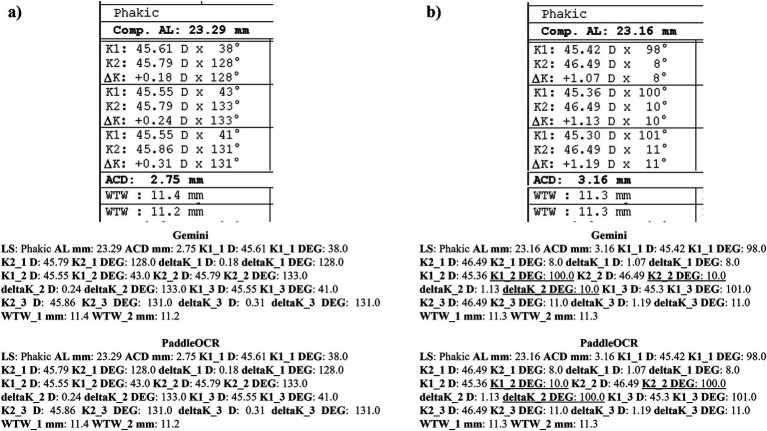
Ocular Biometry OCR for IOLM 500 with results for the **(a)** right eye and **(b)** left eye. For PaddleOCR, the left eye had swapped values for K1_2 DEG, K2_2 DEG, and deltaK_2 DEG. Axial length (AL), flat meridian of the cornea (K1), steep meridian of the cornea (K2), subtraction of K1 from K2 (delta K), anterior chamber depth (ACD), and white-to-white (WTW). Lens status (LS) is at the top.

**Figure 8:**
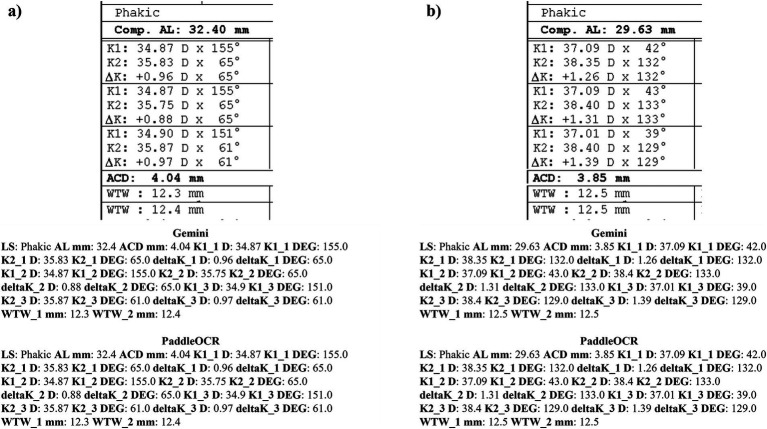
Ocular Biometry OCR for IOLM 500 with results for the **(a)** right eye and **(b)** left eye. Axial length (AL), flat meridian of the cornea (K1), steep meridian of the cornea (K2), subtraction of K1 from K2 (delta K), anterior chamber depth (ACD), and white-to-white (WTW). Lens status (LS) is at the top.

Reviewing the detection scores of PaddleOCR and Gemini, the models achieved uniform predictions across all biometric information. This was supported by the average Cohen-Kappa statistic achieving perfect agreement (+1) for detection. Identified by lower similarity scores, there were a few disputes between PaddleOCR and Gemini for K1 DEG, K2 DEG and deltaK DEG. For these samples, Gemini provided correct predictions while PaddleOCR provided incorrect predictions as verified by the ground truth (Figures 6–8). The PaddleOCR mistakes were likely propagated through the rule checking for angles. Across all three optical biometers, PaddleOCR and Gemini had the highest agreement score for IOLM 500, likely due to the relative simplicity of the biometric data.

### IOLM 700

Assessing the detection scores of PaddleOCR and Gemini, both models’ predictions were near uniform across all metrics. Further, the average Cohen-Kappa statistic implied nearly perfect agreement (0.81–0.99), though there was a significant decline for LS which achieved substantial agreement (0.61–0.80) and VS which achieved fair agreement (0.21–0.40) as a result of the high number of disputed samples (i.e., false negatives and false positives overwhelming true negatives) for detection.

**Figure 9:**
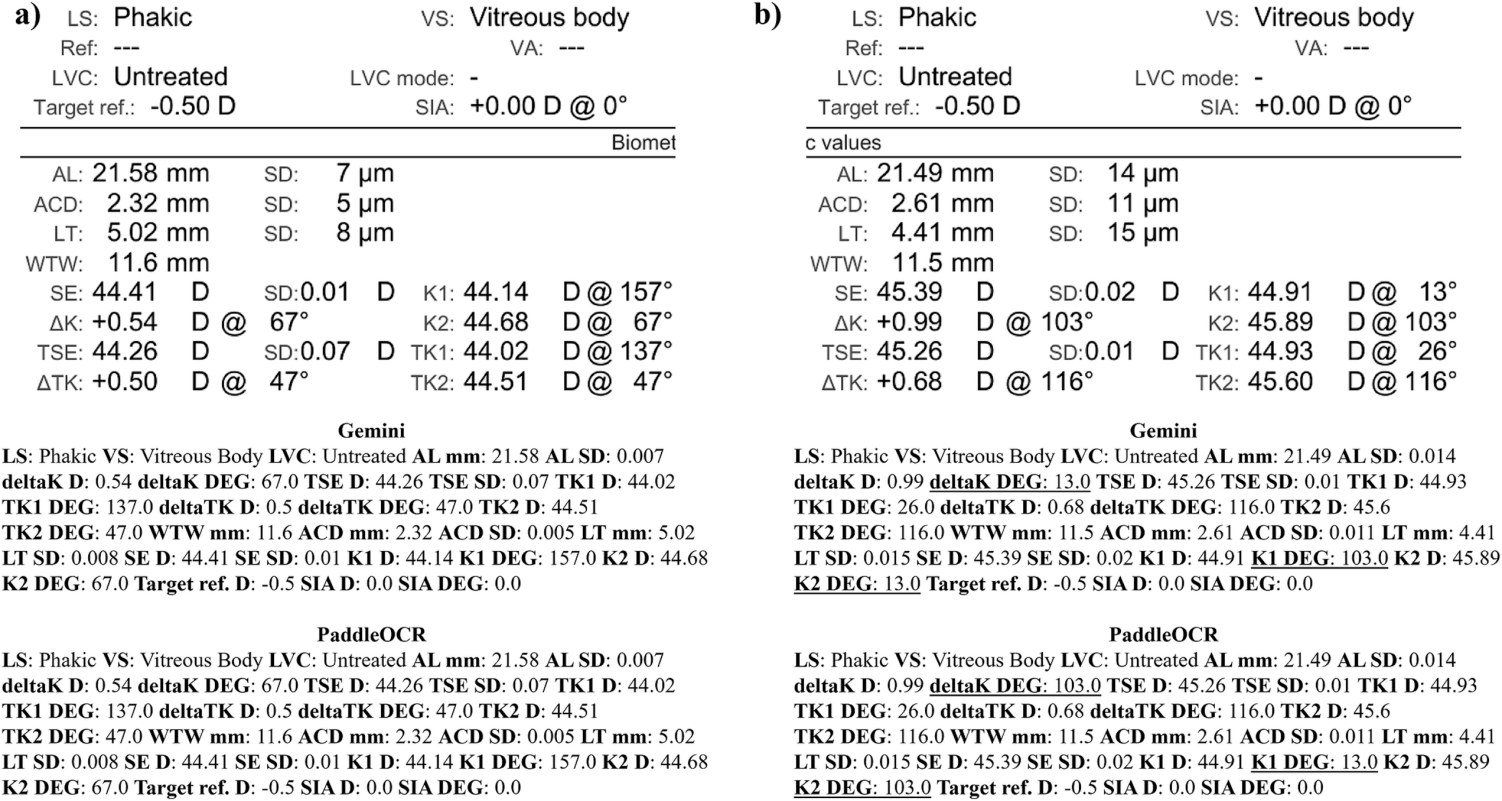
Ocular Biometry OCR for IOLM 700 with results for the **(a)** right eye and **(b)** left eye. For Gemini, the left eye had swapped values for K1 DEG, K2 DEG, and deltaK DEG. Lens status (LS), vitreous status (VS), laser vision correction (LVC), surgically induced astigmatism (SIA), target refraction (Target ref.), axial length (AL), anterior chamber depth (ACD), lens thickness (LT), white-to-white (WTW), spherical equivalent of corneal power according to keratometry (SE), flat meridian of the cornea (K1), steep meridian of the cornea (K2), subtraction of K1 from K2 (delta K), true flat meridian of the cornea (TK1), true steep meridian of the cornea (TK2), subtraction of TK1 from TK2 (delta TK), and spherical equivalent of corneal power according to TK (TSE). Standard deviation (SD) is listed to the side.

**Figure 10:**
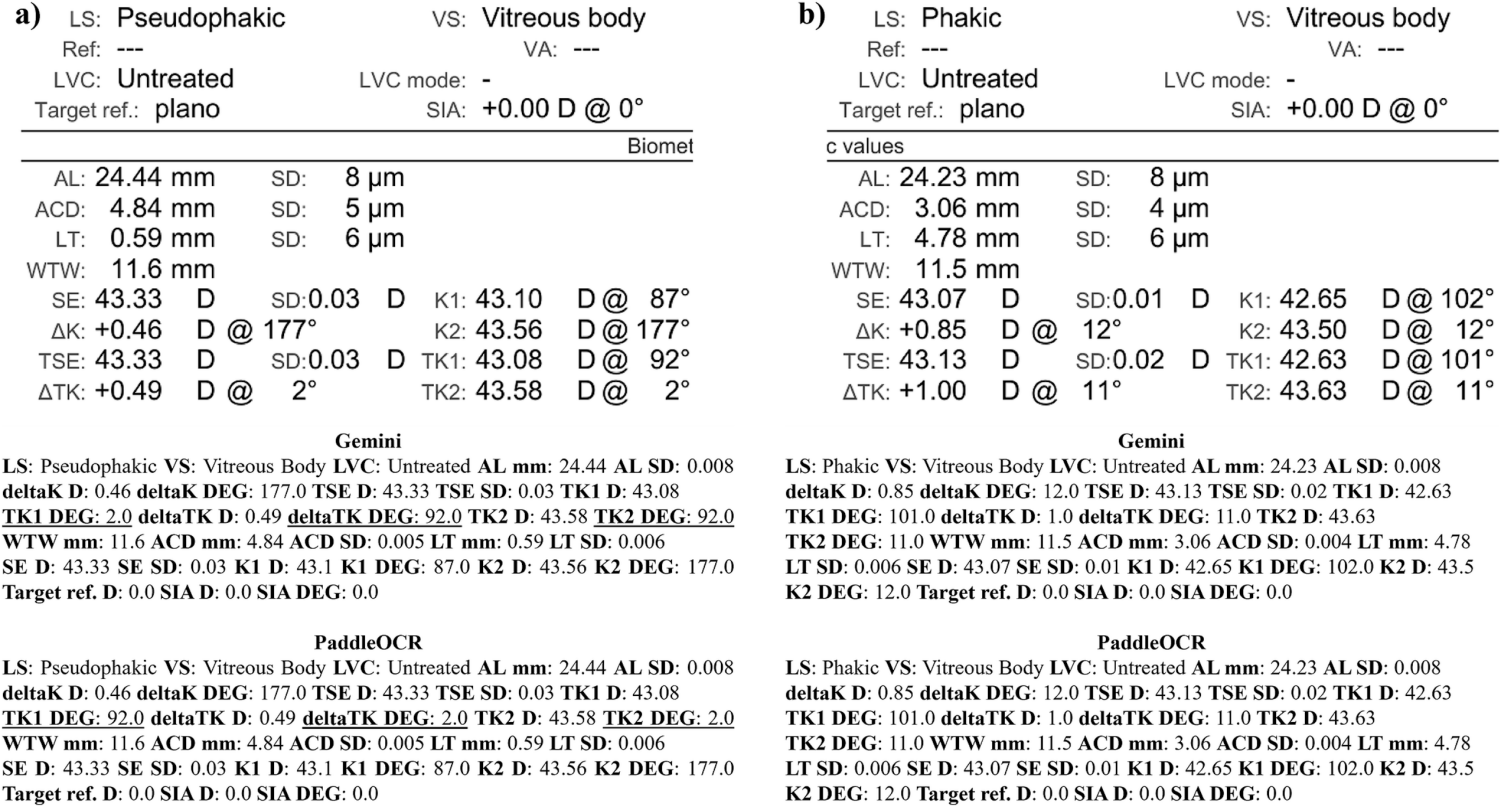
Ocular Biometry OCR for IOLM 700 with results for the **(a)** right eye and **(b)** left eye. For Gemini, the right eye had swapped values for TK1 DEG, TK2 DEG, and deltaTK DEG. Plano for Target ref. is equivalent to 0. Lens status (LS), vitreous status (VS), laser vision correction (LVC), surgically induced astigmatism (SIA), target refraction (Target ref.), axial length (AL), anterior chamber depth (ACD), lens thickness (LT), white-to-white (WTW), spherical equivalent of corneal power according to keratometry (SE), flat meridian of the cornea (K1), steep meridian of the cornea (K2), subtraction of K1 from K2 (delta K), true flat meridian of the cornea (TK1), true steep meridian of the cornea (TK2), subtraction of TK1 from TK2 (delta TK), and spherical equivalent of corneal power according to TK (TSE). Standard deviation (SD) is listed to the side.

**Figure 11:**
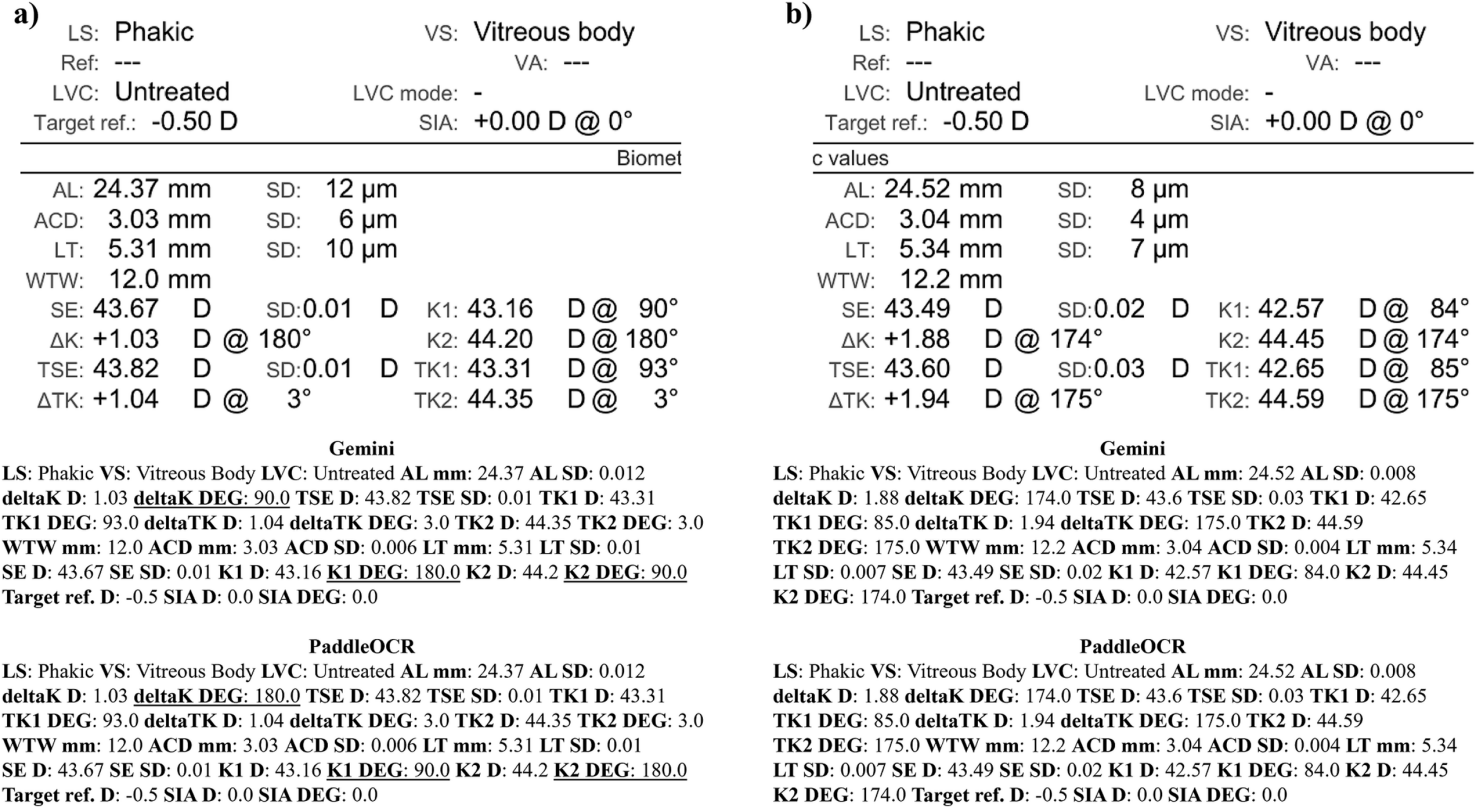
Ocular Biometry OCR for IOLM 700 with results for the **(a)** right eye and **(b)** left eye. For Gemini, the right eye had swapped values for K1 DEG, K2 DEG, and deltaK DEG. Lens status (LS), vitreous status (VS), laser vision correction (LVC), surgically induced astigmatism (SIA), target refraction (Target ref.), axial length (AL), anterior chamber depth (ACD), lens thickness (LT), white-to-white (WTW), spherical equivalent of corneal power according to keratometry (SE), flat meridian of the cornea (K1), steep meridian of the cornea (K2), subtraction of K1 from K2 (delta K), true flat meridian of the cornea (TK1), true steep meridian of the cornea (TK2), subtraction of TK1 from TK2 (delta TK), and spherical equivalent of corneal power according to TK (TSE). Standard deviation (SD) is listed to the side.

Checking the similarity scores of PaddleOCR and Gemini, the metrics that had lower scores included K1 D, K2 D, and deltaK DEG. Reviewing a few of the disputed samples, Gemini had occasionally switched K1 and K2 DEG, an error that would have been propagated by angle rule checking to deltaK DEG (Figures 9–11). The agreement score for IOLM 700 was much higher than that of Lenstar, but just below IOLM 500.

### PaddleOCR and Gemini

As part of the text extraction process, significant steps were taken to address the common mistakes of PaddleOCR and Gemini. A significant drawback of PaddleOCR was incorrectness, especially regarding the angles. The model confused the degree symbol with the number zero. Occasionally, the model also missed the decimal points of values. Hence, the implementation of the rules-based corrections for angles and the counting of per window errors of angles and outlier quantities; however, some errors still managed to trickle through. While the rules-based corrections currently apply to model specific outputs, implementing a similar policy across modalities (PaddleOCR and Gemini) for the same sample may reduce the degree/zero confusion, akin to aggregating predictions.

On the other hand, Gemini had little issue extracting the correct answer; however, the model occasionally provided text in the incorrect order (not left to right, top to bottom) despite being instructed to do so, and substituted missing values with values of other metrics. Indeed, during pipeline development, it was observed that Gemini can hallucinate, especially if a significant amount of data was missing from the report. Accounting for these drawbacks as well considering the wide variation for IOLM 700 reports, the text extraction algorithm was run on multiple windows of varying shape, manually decided by checking the location of the biometric data. Better prompt engineering may also reduce hallucinations; for example, prompting: ‘Extract all the text line-by-line from top-to-bottom, left-to-right normal English formatting’.

Both PaddleOCR and Gemini have their own trade-offs. As a publicly available model, PaddleOCR struggles with accurately identifying metrics and corresponding values underneath specific circumstances but still provides the standard formatting. As a pay for service model, Gemini accurately identifies metrics and corresponding values but struggles at times to provide the normal formatting necessary for useful text extraction. Apart from better fine-tuning of post-processing methods, future studies would involve reducing Gemini’s temperature parameter to see if that would reduce formatting related errors and ensure that results for samples with missing information are consistent. After achieving sufficient text extraction performance with Ocular Biometry OCR, the future aims are: (1) classification of patients to prognosticate RT/RD development via gradient boosted trees and (2) exploratory analysis of clinically relevant attributes associated with classifier decision making.

Apart from use as predictors in ML-tasks, the reports provided by the ubiquitous optical biometers (Lenstar, IOLM 500, IOLM 700) are an informative prerequisite to the most common surgery in the world, cataract surgery (Lee et al., 2008). Ocular Biometry OCR can provide the anatomical information confined within these reports in the form of machine encoded text to enable statistical analysis without requiring manual entry of biometer results. Overall, Ocular Biometry OCR is well generalizable for the aforementioned biometers to which the post-processing methods have been designed to handle the models’ predictions. However, the text extraction pipeline will not generalize to other optical biometer reports because the formatting of the models’ predictions may be different; though ways to adapt Ocular Biometry OCR include selecting new image windows and listing out the metrics of interest.

## Conclusion

In sum, Ocular Biometry OCR, a text extraction pipeline built on PaddleOCR and Gemini, was implemented to collect biometric information from patient IOL reports. The main motivation for not only performing text extraction, but also text post-processing was to ensure the biometric measurements could be semi-automatically put into a usable, tabular format ideal for running ML experiments. The features derived from the biometry dataset will enable the training of downstream ML classifiers.

## Data Availability

The data analyzed in this study is subject to the following licenses/restrictions: must have approval to access high risk Stanford University patient data. Requests to access these datasets should be directed to caludwig@stanford.edu.
